# Expectancy and value beliefs predicting generative AI use: evidence from Chinese university faculty

**DOI:** 10.3389/fpsyg.2026.1758074

**Published:** 2026-04-24

**Authors:** Peng Sun, Yuchi Zhang, Xianmin Yang

**Affiliations:** 1 Human Resources Department, Yancheng Teachers University, Yancheng, China; 2 Department of Educational Technology, School of Smart Education, Jiangsu Normal University, Xuzhou, China

**Keywords:** behavioral intention, generative artificial intelligence, self-efficacy, university teachers, values

## Abstract

**Background:**

Guided by expectancy–value theory, this study investigated how Chinese university teachers’ Generative AI (Gen AI)–specific task values (intrinsic, utility, attainment), perceived Gen AI costs, and Gen AI self-efficacy predict their behavioral intention and frequency of Gen AI use.

**Methods:**

From two universities in China, 365 faculty members completed an online survey.

**Results:**

Structural equation modeling revealed that utility value and self-efficacy positively predicted behavioral intention, while only self-efficacy was a significant predictor of actual usage frequency. Task values and perceived cost, aside from utility value, did not significantly influence outcomes.

**Conclusion:**

These findings highlight the central role of self-efficacy and perceived utility in motivating teachers’ adoption of Gen AI, offering theoretical insights for expectancy–value research and practical guidance for professional development initiatives aimed at fostering effective integration of AI in higher education.

## 1 Introduction

In recent years, generative artificial intelligence (Gen AI) has been a topic of extensive research, with increasing application in the education sector globally (Amofa et al., 2025; Habib, 2025; López-Chila et al., 2024; Montes and Elizondo-Garcia, 2025; Verano-Tacoronte et al., 2025). However, features of Gen AI such as high efficiency, immediacy, and adaptive feedback have created both opportunities and challenges for faculty members in higher education. While studies have recognized behavioral intention and actual frequency of use as key outcomes in assessing educators’ engagement with Gen AI, most studies have relied on technology acceptance frameworks, particularly the Technology Acceptance Model (TAM), to explain the relationships linking attitudes, behavioral intention, and actual use of Gen AI (Xue et al., 2025). While the findings are informative, the TAM perspective overlooks the potential influence of motivational beliefs, such as task values and self-efficacy, which are central constructs in expectancy-value theory (EVT) and provide a more nuanced account of human behavior (Eccles and Wigfield, 2002).

China is one of the most proactive nations in promoting AI’s application in education, with policies explicitly encouraging higher education faculty to integrate Gen AI into teaching and research practices (e.g., State Council of the People's Republic of China, 2025). In this policy-driven context, motivational beliefs of faculty members could play a decisive role in translating opportunities into actual adoption behaviors.

This study, therefore, draws on EVT to examine how university teachers’ behavioral intentions and frequency of Gen AI use are shaped by their (a) perceptions of the task values and cost and (b) their self-efficacy in using Gen AI. By extending the theoretical lens beyond technology acceptance models, this study seeks to understand the motivational mechanisms underlying Gen AI adoption by teachers in higher education and contribute practical insights for fostering effective integration of Gen AI in their teaching.

### 1.1 Expectancy-value theory

The expectancy–value theory (EVT) provides a rudimentary framework for explaining why individuals choose, persist in, or withdraw from tasks by balancing their expectations for success against the perceived task values and costs (Eccles and Wigfield, 2002). According to EVT, expectancy–value–cost beliefs can predict both behavioral intentions and actual behaviors. These beliefs are typically reflected in task values (intrinsic, utility, and attainment), perceived costs, and self-efficacy (Eccles and Wigfield, 2002).

Intrinsic value refers to the extent to which individuals find a task enjoyable or interesting in itself (Eccles and Wigfield, 2002). A higher intrinsic value has been found associated with stronger learning motivation, greater technology adoption, and higher engagement in knowledge acquisition (Jiang et al., 2018; Lee and Song, 2022; Plante et al., 2013; Yin and Goh, 2025). In the higher education context, when teachers perceive Gen AI as enjoyable or stimulating, their intention to use it in their work and their frequency of use are both high.

Utility value refers to the instrumental usefulness of a task for achieving current or future goals, such as improving teaching efficiency or advancing academic careers. University teachers develop stronger usage intentions and higher levels of actual use if they realize that Gen AI can enhance their administrative, teaching, or research tasks (Kuhn et al., 2022).

Attainment value refers to the importance of a task for one’s self-concept and professional identity (Eccles and Wigfield, 2002). China’s government policies strongly encourage higher education faculty to explore and apply Gen AI in teaching and research (State Council of the People's Republic of China, 2025). Thus, when teachers perceive Gen AI use aligning with their professional competence and identity, their motivation to adopt it and to use it more extensively is higher.

In contrast, cost refers to the perceived negative aspects of task engagement, such as time, effort, cognitive load, or opportunity costs (Eccles and Wigfield, 2002; Flake et al., 2015). Teachers who view Gen AI as overly demanding or resource-intensive are less likely to integrate it into their professional practices.

Finally, self-efficacy, emphasized both in EVT and social cognitive theory, reflects individuals’ confidence in their ability to complete a task successfully. When Gen AI self-efficacy of teachers is high, they are more likely to have stronger behavioral intentions and actually use Gen AI more frequently (Eccles and Wigfield, 2002).

Despite its explanatory power, the EVT framework has faced criticism for its predominantly cognitive focus. Theoretical reviews suggest that EVT posits individuals as rational decision-makers who meticulously calculate costs and benefits, often overlooking the fact that human behavior frequently violates economic assumptions of rationality (Steel and König, 2006). This rationalist bias tends to neglect the immediate influence of non-cognitive factors—such as affective reactions or “technology anxiety”—that are crucial in educational and technological contexts (Wang et al., 2024). Particularly regarding anthropomorphic or disruptive technologies like Gen AI, recent studies highlight that user acceptance is not solely a calculation of utility but is deeply intertwined with psychological paradoxes and anxiety (Zhu et al., 2023). Recognizing this limitation, the present study explicitly emphasizes perceived cost not merely as an investment of time, but as a construct capturing the cognitive burden and psychological barriers associated with AI adoption. Furthermore, we position Self-efficacy as a critical bridge, addressing the confidence gap that rational value beliefs alone cannot bridge.

However, studies on how these motivational beliefs shape Gen AI adoption among university teachers are limited, with no empirical research so far directly examining the relationships linking these five factors and teachers’ Gen AI-related outcomes. The only related study in the literature (Yin and Goh, 2025) focused on adult learners, 609 university students, and found that all three types of task value promoted students’ intention to learn and use Gen AI, while perceived cost was negatively associated with Gen AI–related outcomes. On one hand, the pragmatic orientation driven by evaluation metrics suggests that utility Value—the extent to which Gen AI enhances research and teaching efficiency—could be a dominant motivator for faculty coping with time scarcity (Steel and König, 2006). On the other hand, Chinese culture also places a high premium on the “teacher as a scholar-innovator” identity. Consequently, attainment value (professional identity) and intrinsic value (intellectual curiosity) remain theoretically vital, as they drive teachers to embrace AI not just for survival, but for professional self-actualization. Furthermore, given the disruptive nature of Gen AI, teachers likely face “paradoxical expectations”: they may oscillate between perceiving AI as a helpful assistant (Utility) and fearing it as a source of complexity or replacement (cost/anxiety) (Zhu et al., 2023). Therefore, examining how these diverse value beliefs and perceived costs jointly—or differentially—shape adoption in this high-pressure context is critical.

### 1.2 The structural and cultural context

To fully understand the motivational dynamics of Chinese university teachers, it is essential to consider the specific structural and cultural context. Currently, China’s higher education system operates under a “dual-drive” mechanism: high-stakes, performance-based accountability reforms (e.g., the ‘Double First-Class’ initiative) paralleled by a vigorous promotion of educational digitalization (State Council of the People's Republic of China, 2025; Zheng and Li, 2025). Specifically, the “Double First-Class” initiative links institutional funding and faculty career advancement to quantifiable research metrics, creating a high-pressure environment for academic productivity. Simultaneously, national digitalization strategies position the integration of AI not merely as a pedagogical choice, but as a policy imperative for modernizing higher education.

In this unique ecosystem, the components of EVT may manifest with distinct intensities. On one hand, the instrumental rationality driven by evaluation metrics suggests that Utility Value—the extent to which Gen AI enhances research and teaching efficiency—could be a dominant motivator for faculty coping with time scarcity (Steel and König, 2006). On the other hand, Chinese culture also places a high premium on the “teacher as a scholar-innovator” identity. The Confucian tradition traditionally reveres the teacher as the “owner of knowledge” (Yu, 1996), creating a deep-seated professional identity rooted in scholarship. Indeed, attaining scholarship (*liyan*) is historically viewed as a paramount form of achievement and a path to immortality in the Confucian value system (Yu, 1996). Structurally, the “Double First-Class” initiative has further accelerated the transformation of faculty identity toward becoming “productive researchers” (State Council of the People's Republic of China, 2025). Thus, the “teacher as a scholar-innovator” identity remains a powerful motivational force, theoretically supporting the relevance of attainment and intrinsic values even within a high-pressure context. Consequently, attainment value (professional identity) and intrinsic value (intellectual curiosity) remain theoretically vital, as they drive teachers to embrace AI not just for survival, but for professional self-actualization. Furthermore, given the disruptive nature of Gen AI, teachers likely face paradoxical expectations: they may oscillate between perceiving AI as a helpful assistant (utility) and fearing it as a source of complexity or replacement (cost/anxiety) (Zhu et al., 2023). Therefore, examining how these diverse value beliefs and perceived costs jointly—or differentially—shape adoption in this high-pressure context is critical.

### 1.3 The present study

University teachers play a pivotal role in shaping the attitudes of future generations toward artificial intelligence and guiding them in its appropriate use in academic and professional contexts. As both educators and researchers, they influence students’ technological engagement and contribute to the advancement of scientific innovation (Xue et al., 2025). Consequently, understanding the factors that motivate teachers to adopt Gen AI can be of substantial theoretical and practical significance (Aljuaid, 2024; d'Amato et al., 2025; Divino, 2024; Verano-Tacoronte et al., 2025).

AI adoption in education is well-researched in the literature; however, little attention has been paid to how teachers’ expectancy–value–cost beliefs specifically shape their Gen AI–related outcomes (Terblanche et al., 2023) or the broader motivational beliefs that underpin teachers’ behavioral intentions and actual usage. This gap restricts our understanding of why some teachers actively integrate Gen AI into their work while others remain reluctant. To address this issue, a cross-sectional design is adopted, and structural equation modeling (SEM) is employed, in this study to examine how task values (intrinsic, utility, attainment), perceived costs, and self-efficacy jointly predict behavioral intention and usage frequency of Gen AI among university teachers in China.

**Figure 1:**
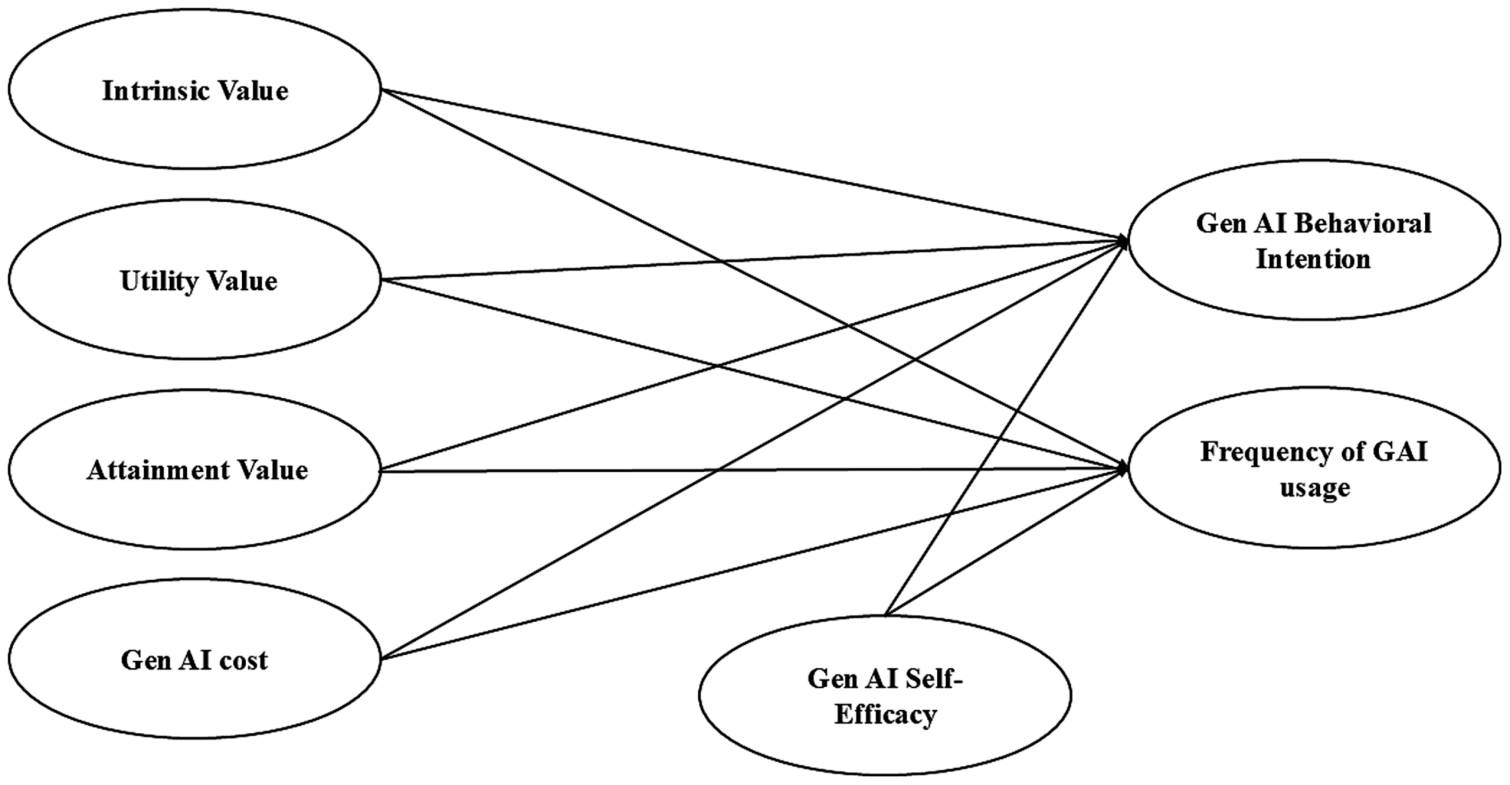
Hypothesized conceptual model.

Both behavioral intention and usage frequency were included as outcome variables because intention and actual use represent conceptually distinct, yet complementary, facets of technology adoption. While intention captures teachers’ motivational readiness and evaluative stance toward engaging with Gen AI, frequency of use reflects the enacted behavior in actual professional contexts, which may be constrained or facilitated by institutional policies, available resources, or time pressures. Measuring both avoids over-relying on the intention–behavior link and helps recognize that strong intentions do not always translate into frequent usage. By modeling them simultaneously (see Figure 1), this study provides a more comprehensive account of how expectancy–value–cost beliefs and self-efficacy shape both the willingness to adopt Gen AI and the extent to which it becomes integrated into teachers’ daily academic and teaching practices.

Based on expectancy–value theory and prior empirical evidence, the following hypotheses are proposed:

*H1*: Intrinsic value, utility value, and attainment value are positively associated with teachers’ Gen AI behavioral intention and usage frequency.

*H2*: Perceived cost is negatively associated with teachers’ Gen AI behavioral intention and usage frequency.

*H3*: Self-efficacy is positively associated with teachers’ Gen AI behavioral intention and usage frequency.

## 2 Materials and methods

### 2.1 Participants and procedure

This study was conducted in accordance with the Declaration of Helsinki (1975, revised in 2013) and was approved by the Institutional Review Board of the corresponding author’s affiliated institution. Participants were 368 full-time faculty members recruited from two comprehensive universities located in Jiangsu Province, China. Recruitment was conducted via internal university communication channels, and participation was voluntary. At the time of the study, all participants were employed as higher education instructors across various disciplines, ensuring representation across a broad range of academic fields.

Data collection was through an online survey platform. Participants were informed of the purpose of the study, assured of the confidentiality of their responses, and were asked to provide their informed consent electronically before accessing the questionnaire. The survey required approximately 15 min to complete. To ensure data quality, response patterns were screened for irregularities. Three cases showing patterned responses were removed from the dataset, leaving 365 valid responses for the final analysis (Table 1).

**Table 1:** Demographic characteristics of the sample.

Characteristic	Category	*n*
Sex	Female	190
	Male	175
Teaching experience	Less than 5 years	71
	6–10 years	112
	11–20 years	118
	21–30 years	48
	More than 30 years	16
Academic title	Lecturer	118
	Associate Professor	114
	Professor	73
Positions	Teaching and Research Position	147
	Full-Time Research Position	37
	Full-Time Teaching Position	97
	Counselor Position	23
	Administrative Position	34
	Laboratory/Technical Position	25
	Other	2

### 2.2 Measures

#### 2.2.1 Gen AI task values

Participants’ task values regarding Gen AI were measured using a modified version of the expectancy-value framework developed by Wigfield and Eccles (2000), adapted to reflect the professional context of Chinese university faculty (Cheng et al., 2020). The Gen AI Task Value scale comprised 16 items in total, categorized into three subdimensions: intrinsic value (5 items), utility value (6 items), and attainment value (5 items). Each item was rated on a 7-point Likert scale (1 = strongly disagree, 7 = strongly agree). (1) Intrinsic Value. This subdimension captures the degree to which participants find using Gen AI inherently interesting or enjoyable. Example item: “I find using Gen AI for teaching very interesting.” (2) Utility Value. This subdimension reflects the perceived usefulness of Gen AI in achieving professional goals. Example item: “Using Gen AI helps me complete teaching tasks more efficiently.” (3) Attainment Value. This subdimension measures the extent to which using Gen AI aligns with one’s professional identity and important goals. Example item: “It is important for me to use Gen AI in my work.” Using this scale helped capture faculty members’ subjective valuations of Gen AI across different motivational dimensions, providing a comprehensive assessment of how task values may influence both behavioral intention and usage frequency. The internal consistency reliabilities (Cronbach’s α) for the three subscales were as follows: Intrinsic Value (α = 0.83), Utility Value (α = 0.88), and Attainment Value (α = 0.86).

#### 2.2.2 Gen AI self-efficacy

This construct was measured using a modified version of the Technology Self-Efficacy scale to fit the context of this study (Saville and Foster, 2021). Participants rated their agreement with five items on a 7-point Likert scale (1 = Strongly disagree, 7 = Strongly agree). A sample item was: “I am fully confident in my ability to use Gen AI technology to enhance teaching quality.” Cronbach’s alpha for this scale was 0.82.

#### 2.2.3 Gen AI cost

Gen AI cost was measured using an adapted scale from prior research (Flake et al., 2015). The scale consisted of five items rated on a 7-point Likert scale (1 = strongly disagree, 7 = strongly agree). An example item was as follows: “I have too many other responsibilities to put enough effort into learning and using Gen AI.” Cronbach’s alpha for this scale was 0.81.

#### 2.2.4 Gen AI behavioral intention

Participants’ intention to use Gen AI was measured using a modified version of a previously validated Behavioral Intention scale, adapted to fit the context of this study (Xu et al., 2024). The original items (e.g., “I will strive to use AI tools in my daily life”) were revised to explicitly reference Gen AI and higher education contexts (e.g., “I will strive to use Gen AI tools in my daily teaching and research”). Participants rated their agreement with five items on a 7-point Likert scale (1 = Strongly disagree, 7 = Strongly agree). Cronbach’s alpha for this scale was 0.83.

#### 2.2.5 Frequency of Gen AI usage

A single-item measure was used to assess the frequency of Gen AI usage. Participants were asked, “Have you used Gen AI (e.g., ChatGPT)?” and were asked to respond on a 7-point Likert scale ranging from 1 (Never) to 7 (Multiple times per day).

### 2.3 Data analysis plan

Data were processed and analyzed using SPSS 26 and AMOS 26. Given that the scales were applied in mainland China for the first time, the first author translated the original items into Mandarin, followed by back-translation into English by the corresponding author. Item wording was refined based on semantic equivalence to ensure accuracy. Next, Confirmatory Factor Analysis (CFA) was performed to assess the construct validity of the scales. Model fit was evaluated using multiple indices: Chi-square/df (*χ*^2^/*df*), Goodness-of-Fit Index (GFI ≥ 0.90), Incremental Fit Index (IFI ≥ 0.95), Tucker-Lewis Index (TLI ≥ 0.95), Comparative Fit Index (CFI ≥ 0.95), and Root Mean Square Error of Approximation (RMSEA ≤ 0.06) (Hu and Bentler, 1999), consistent with established fit criteria. Finally, descriptive statistics and Pearson correlation analyses were conducted in SPSS 26 to summarize the data distribution and examine relationships among the key variables. Subsequently, SEM was performed to test the hypothesized paths. To ensure the robustness of the findings and control for potential confounding effects, sex and teaching experience were incorporated into the structural model as covariates, based on their significant associations with the outcome variables in the preliminary correlation analyses.

## 3 Results

### 3.1 Preliminary analyses

The correlation analysis results showed that the three types of Gen AI task values and Gen AI self-efficacy were significantly positively correlated with Gen AI behavioral intention, whereas Gen AI cost was significantly negatively correlated with behavioral intention. This negative association was theoretically anticipated, as higher scores on the Gen AI Cost scale reflected greater perceived burdens (i.e., barriers). Only attainment value and Gen AI self-efficacy demonstrated weak but significant positive correlations with the frequency of Gen AI usage (Table 2).

**Table 2:** Descriptive statistics correlation coefficient matrix (*N* = 368).

	1	2	3	4	5	6	7
1. Intrinsic value	**0.71**						
2. Utility value	0.49	**0.75**					
3. Attainment value	0.52	0.55	**0.71**				
4. Gen AI cost	−0.40	−0.43	−0.45	**0.69**			
5. Gen AI self-efficacy	0.57	0.53	0.60	−0.45	**0.65**		
6. Gen AI behavioral intention	0.47	0.51	0.51	0.34	0.52	**0.75**	
7. Frequency of Gen AI usage	0.06	0.09	0.14	−0.39	0.19	0.05	–
*M*	4.60	4.61	4.66	3.50	4.57	4.83	5.67
*SD*	1.45	1.47	1.50	1.40	1.44	1.48	2.83

All correlations were significant (*p* < 0.05). Diagonal elements (in bold) represent the square root of the Average Variance Extracted (AVE). Off-diagonal elements represent the Pearson correlations between constructs. Gen AI Usage Frequency was measured by a single item, AVE statistics are not applicable.

### 3.2 Measurement model assessment

The assessment The psychometric properties of the measurement scales were evaluated through Confirmatory Factor Analysis (CFA) to ensure construct validity and reliability prior to testing the structural relationships. The measurement model specified six latent constructs: intrinsic value, utility value, attainment value, Gen AI cost, Gen AI self-efficacy, and behavioral intention. Each observed item was constrained to load onto its respective theoretical factor, and all latent variables were allowed to covary freely.

The assessment revealed robust item quality and satisfactory reliability. Standardized factor loadings for all items were statistically significant (*p* < 0.001) and exceeded the recommended threshold of 0.45 (Hu and Bentler, 1999), ranging from 0.57 to 0.78. Reliability analysis indicated that the Composite Reliability (CR) values for all constructs ranged from 0.78 to 0.88, comfortably exceeding the 0.70 benchmark (Hair et al., 2019). Regarding convergent validity, the Average Variance Extracted (AVE) values for the three task value dimensions were above the 0.50 threshold. Although the AVE values for Gen AI Cost (0.47) and Self-Efficacy (0.42) were marginally below 0.50, their convergent validity was deemed acceptable according to the criteria established by Fornell and Larcker (1981), which state that a construct’s convergent validity is acceptable if the AVE is below 0.50 provided that the CR is higher than 0.60.

To address concerns regarding high correlations among constructs and to ensure discriminant validity, we applied the Fornell-Larcker criterion (Fornell and Larcker, 1981). As presented in Table 3, the square root of the AVE for each construct (bold values on the diagonal, ranging from 0.65 to 0.75) exceeded the highest correlation between that construct and any other latent variable in the model, thereby supporting discriminant validity. Furthermore, given the theoretical relatedness of the value constructs, we explicitly tested for multicollinearity by calculating the Variance Inflation Factor (VIF). The results indicated that multicollinearity was not a concern. The VIF values for all predictors were consistently low: 1.69 for Intrinsic Value, 1.76 for Utility Value, 1.95 for Attainment Value, 1.41 for Gen AI Cost, 2.02 for Gen AI Self-Efficacy, and 1.64 for Gen AI Behavioral Intention. All values were well below the conservative threshold of 3.0 (Hair et al., 2019), confirming that the constructs were empirically distinct and that the structural model estimates were not biased by multicollinearity.

**Table 3:** Psychometric properties of the measurement scales.

Construct/items	Standardized factor loadings	Item-total correlation	Cronbach α	AVE	CR
Intrinsic value			0.83	0.83	0.50
Intrinsic_Item1	0.73	0.77^**^			
Intrinsic_Item2	0.73	0.78^**^			
Intrinsic_Item3	0.66	0.75^**^			
Intrinsic_Item4	0.70	0.78^**^			
Intrinsic_Item5	0.70	0.77^**^			
Utility value			0.88	0.88	0.56
Utility_Item1	0.71	0.77^**^			
Utility_Item2	0.76	0.80^**^			
Utility_Item3	0.75	0.79^**^			
Utility_Item4	0.78	0.82^**^			
Utility_Item5	0.73	0.78^**^			
Utility_Item6	0.77	0.81^**^			
Attainment value			0.86	0.84	0.51
Attainment_Item1	0.65	0.76^**^			
Attainment_Item2	0.75	0.83^**^			
Attainment_Item3	0.73	0.80^**^			
Attainment_Item4	0.72	0.81^**^			
Attainment_Item5	0.72	0.80^**^			
Gen AI cost			0.81	0.81	0.47
Cost_Item1	0.70	0.74^**^			
Cost_Item2	0.70	0.77^**^			
Cost_Item3	0.69	0.77^**^			
Cost_Item4	0.67	0.75^**^			
Cost_Item5	0.70	0.76^**^			
Gen AI self-efficacy			0.82	0.78	0.42
Efficacy_Item1	0.69	0.80^**^			
Efficacy_Item2	0.66	0.77^**^			
Efficacy_Item3	0.57	0.71^**^			
Efficacy_Item4	0.67	0.78^**^			
Efficacy_Item5	0.64	0.76^**^			
Gen AI behavioral intention			0.83	0.57	0.80
Intention_Item1	0.73	0.85^**^			
Intention_Item2	0.77	0.86^**^			
Intention_Item3	0.75	0.88^**^			

AVE, Average Variance Extracted; CR, Composite Reliability. ^**^p < 0.01. Gen AI Usage Frequency was measured by a single item; therefore, CR and AVE statistics are not applicable.

### 3.3 Structural model and hypothesis testing

Following the validation of the measurement model, Structural Equation Modeling (SEM) was performed to test the hypothesized structural relationships. The evaluation of the structural model yielded an excellent fit to the observed data: *χ*^2^ = 699.02, *df* = 445, *p* < 0.001, *χ*^2^ = 2/*df* = 1.57, GFI = 0.90, CFI = 0.95, IFI = 0.95, and RMSEA = 0.04 (90%CI = [0.03, 0.04]). These strong goodness-of-fit indices collectively demonstrated that the proposed theoretical framework provided a robust and adequate account of the associations being studied.

**Figure 2:**
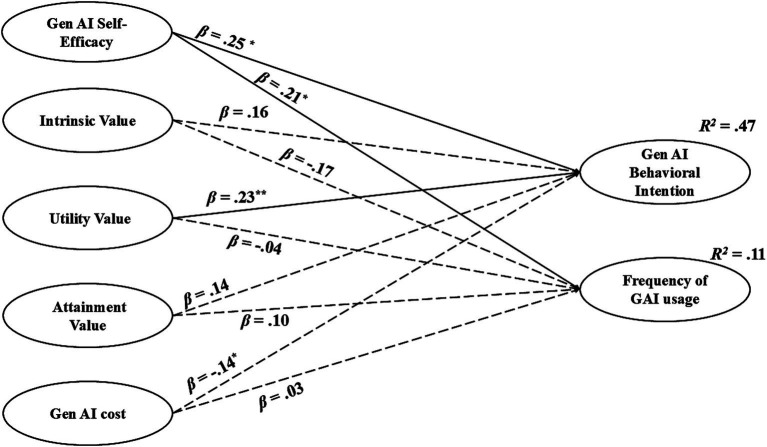
Standardized structural equation model of predictors of Gen AI behavioral intention and frequency of use. *N* = 365. Values presented on the single-headed arrows are standardized path coefficients (β). Solid lines represent statistically significant relationships, whereas dashed lines indicate non-significant paths. To maintain visual clarity, the measurement indicators (items), residual errors, correlations among exogenous latent variables, and demographic control variables (sex and teaching experience) are omitted from this diagram. The values are annotated at the top right of Gen AI behavioral intention and usage frequency (*R*^2^). For full statistical details of all paths and controls, please refer to Table 4. ^*^p < 0.05. ^**^p < 0.01.

To ensure the robustness of the findings and control for potential confounding effects, sex and teaching experience were incorporated into the structural model as demographic covariates, based on their significant associations with the outcome variables in the preliminary analyses. The structural results, detailed in Table 4 and visually summarized in Figure 2, revealed that utility value and Gen AI self-efficacy significantly and positively predicted university teachers’ Gen AI behavioral intention. Notably, Gen AI self-efficacy emerged as the sole significant predictor of actual usage frequency. In contrast, the effects of intrinsic value, attainment value, and perceived cost on both outcome variables did not reach statistical significance. Covariance among the exogenous latent variables was explicitly modeled, confirming that the associations among these motivational constructs were consistent with the Expectancy-Value Theory framework. Even after controlling for the effects of demographic covariates, the core paths of the theoretical model remained significant, confirming the stability and explanatory power of the motivational predictors.

**Table 4:** Structural model path coefficients and hypothesis testing results.

Hypothesis	Path	β	S. E.	*t*	*p*	Result
H1	Intrinsic value → Gen AI behavioral intention	0.16	0.08	1.92	0.06	Not supported
H1	**Utility value → Gen AI behavioral intention**	**0.23**	**0.07**	**2.97**	**0.00**	**Supported**
H1	Attainment value → Gen AI behavioral intention	0.14	0.09	1.59	0.11	Not supported
H1	Intrinsic value → Gen AI usage frequency	−0.17	0.19	−1.85	0.07	Not supported
H1	Utility value → Gen AI usage frequency	−0.04	0.17	−0.54	0.59	Not supported
H1	Attainment value → Gen AI usage frequency	0.10	0.21	1.03	0.30	Not supported
H2	**Perceived cost → Gen AI behavioral intention**	**−0.14**	**0.06**	**−2.17**	**0.03**	**Supported**
H2	Perceived cost → Gen AI usage frequency	0.03	0.15	0.46	0.64	Not supported
H3	**Self-efficacy → Gen AI behavioral intention**	**0.25**	**0.09**	**2.50**	**0.01**	**Supported**
H3	**Self-efficacy → Gen AI usage frequency**	**0.21**	**0.209**	**1.980**	**0.05**	**Supported**
Demographic covariates						
	Sex → Gen AI behavioral intention	0.08	0.12	1.58	0.12	–
	Sex → Gen AI usage frequency	0.09	0.29	1.72	0.09	–
	**Teaching experience → Gen AI behavioral intention**	**0.12**	**0.06**	**1.99**	**0.05**	–
	**Teaching experience → Gen AI usage frequency**	**−0.67**	**0.14**	**−4.71**	**0.00**	–

β = Standardized path coefficients. Bold values indicate path coefficients that are statistically significant at the 0.05 level (two-tailed). Gen AI, Generative Artificial Intelligence.

## 4 Discussion

This study contributes to the growing literature on Gen AI adoption in higher education by highlighting the EVT (Eccles and Wigfield, 2002). While most prior research has relied on technology acceptance frameworks such as the TAM to account for educators’ attitudes, intentions, and use of Gen AI, these models primarily capture evaluative appraisals of technology but provide a limited account of the motivational and belief systems that underlie adoption behaviors (Jogezai et al., 2025; Li et al., 2025). By situating faculty members’ engagement with Gen AI within the EVT framework, this study underscores the importance of task values, perceived costs, and self-efficacy as more fundamental motivational antecedents of adoption. This theoretical shift not only enriches understanding of why educators differ in their willingness and ability to integrate Gen AI into professional practice but also advances the broader literature by bridging technology adoption research with motivational psychology. The structural model demonstrates an excellent fit to the empirical data, as all key goodness-of-fit indices consistently surpass conventional psychometric benchmarks. Such robust alignment suggests that the proposed theoretical framework provides a highly adequate account of the motivational mechanisms underlying Gen AI adoption among university faculty.

The findings showed that utility value significantly predicted teachers’ Gen AI behavioral intention, whereas intrinsic and attainment values did not reach significance. This result partially supported Hypothesis 1. This pattern is broadly compatible with EVT—which predicts that task values drive motivation—but it refines EVT’s expectations by showing that, in this context, the utility dimension is the primary driver of intention while the enjoyment- and identity-based dimensions exert weaker effects (Eccles and Wigfield, 2002).

Several, theoretically grounded, reasons can explain why utility value emerges as the dominant predictor. Utility value directly signals the *instrumentality* of Gen AI for achieving salient, task-relevant goals (e.g., time savings, grading efficiency, and research outputs) (Eccles and Wigfield, 2002). Instrumental benefits are concrete and immediately actionable, thereby providing clear, calculable returns that translate readily into an intention to adopt a tool for work-related tasks. In organizational or policy-driven settings—where performance metrics, workload, and efficiency are foregrounded—the perceived usefulness of a technology typically exerts a stronger motivational pull than more diffuse sources of motivation (State Council of the People's Republic of China, 2025). Empirical work on technology adoption and teacher motivation similarly shows that perceived usefulness or task-relevance often predicts adoption more robustly than interest-based motives, particularly when adoption carries clear productivity gains (Backfisch et al., 2024; Thompson-Lee et al., 2024; Yin and Goh, 2025).

The prominence of utility value in predicting behavioral intention echoes the specific structural and cultural context identified in the introduction. Reflecting the academic environment observed and experienced by the researchers, Chinese faculty members operate within a “dual-drive” ecosystem characterized by high-stakes performance accountability (e.g., the “Double First-Class” initiative) and intensified time scarcity (State Council of the People's Republic of China, 2025; Steel and König, 2006). In such a policy-intensive environment, the adoption of Gen AI appears to be governed less by intrinsic interest and more by a pragmatic orientation toward efficiency. Specifically, the pressure to meet quantifiable research and teaching metrics likely compels teachers to prioritize tools that offer immediate solutions to workload challenges. Thus, utility value—the perception of Gen AI as an effective aid for survival and productivity—emerges as the dominant motivational force, overriding the need for mere intellectual exploration. This pattern highlights a prevailing tendency to prioritize the practical utility of Gen AI, reflecting a pragmatic adaptation to the digitalization of higher education in China.

By contrast, intrinsic and attainment values may have been insufficient to predict intention in this sample for several nonexclusive reasons. First, in a policy-intensive and evaluation-oriented environment, pragmatic considerations may eclipse enjoyment- or identity-based motives, rendering intrinsic enjoyment and identity-enhancement less decisive for immediate adoption decisions (Eccles and Wigfield, 2002). Second, early adopters’ use may be driven by perceived utility, whereas intrinsic enjoyment may emerge only later as users become more proficient and experientially rewarded; thus intrinsic effects may be delayed and better captured in longitudinal designs. Consistent with prior evidence on employment-related tasks, motivation for externally mandated or evaluative tasks tends to be extrinsic rather than intrinsic (Remedios and Sewell, 2024). In the context of higher education, Chinese university teachers’ engagement with Gen AI may similarly reflect a policy-driven or job-required obligation, which could explain why intrinsic and attainment values did not significantly predict behavioral intention or usage frequency in this study.

Therefore, to summarize, the results suggest that, for university faculty operating within a performance- and policy-driven context, emphasizing the *usefulness* of Gen AI is likely to be more effective in shaping adoption intentions than appeals to enjoyment or professional identity—although intrinsic and attainment values may still matter under different conditions (e.g., later adoption stages, stronger institutional incentives) or via indirect pathways. Future work should therefore test contextual moderators (policy pressure, reward structures) and temporal dynamics to establish when and for whom intrinsic and attainment values become consequential.

The findings revealed that perceived cost did not significantly influence either behavioral intention or frequency of Gen AI use, which does not support Hypothesis 2. A plausible explanation lies in the developmental stage of Gen AI adoption among the faculty in this sample. As university teachers are still in the early phase of exploring and experimenting with Gen AI, they may not yet fully experience the potential burdens often associated with sustained technology integration, such as time demands, cognitive load, or opportunity costs. Consequently, the perceived costs of Gen AI use may remain relatively low or less salient compared to its perceived benefits, thereby attenuating their predictive power in shaping teachers’ motivational and behavioral outcomes.

The results further demonstrated that Gen AI self-efficacy significantly predicted both behavioral intention and actual frequency of use, thereby supporting Hypothesis 3. Within the EVT framework, self-efficacy reflects the expectancy component, representing individuals’ confidence in their ability to successfully accomplish a task (Eccles and Wigfield, 2002). When faculty members perceive themselves as competent in mastering and applying Gen AI, they are more likely to form strong intentions to engage with the technology and, crucially, to translate these intentions into actual usage. This dual influence underscores the central role of expectancy beliefs in bridging the gap between motivational readiness and enacted behavior, highlighting that confidence in one’s capability is not only motivational but also instrumental in enabling the consistent integration of Gen AI into professional practice (Zeeb and Voss, 2025).

These findings indicate a partial dissociation between predictors of behavioral intention and actual usage frequency, with only Gen AI self-efficacy consistently influencing both outcomes. This pattern suggests the potential presence of an intention–behavior gap, whereby strong intentions do not always translate into frequent use. Future research could employ person-centered approaches, such as latent profile analysis (LPA), to examine heterogeneous patterns of intention and usage among faculty (Laursen and Hoff, 2006). Such analyses would provide nuanced insights into the diverse ways in which teachers engage with Gen AI and offer empirical guidance for designing targeted training programs that promote more consistent and effective technology adoption.

## 5 Conclusion

In an era where Gen AI increasingly shapes teaching practices and student learning, understanding the antecedents of university teachers’ Gen AI-related outcomes from the perspective of motivation and beliefs represents a crucial step toward developing a human behavioral science attuned to the Gen AI era (Bonnefon et al., 2024; Tsvetkova et al., 2024; Wan et al., 2025; Wijaya et al., 2024). This study aimed to examine how university teachers’ task values, perceived costs, and self-efficacy shape their behavioral intentions and actual frequency of Gen AI use, drawing on the EVT framework (Eccles and Wigfield, 2002). The study makes a theoretical contribution by extending EVT to a novel technology adoption context in higher education and by demonstrating the differential roles of motivational beliefs in predicting intention versus enacted behavior. The practical contribution of the study is that its findings provide evidence for designing targeted professional development programs that enhance teachers’ Gen AI self-efficacy and underscore the utility value of Gen AI in teaching. Nonetheless, the study has limitations as well; these include its cross-sectional design, which precludes causal inference, and the restricted representativeness of the sample, drawn from only two universities in one province of China. Future research should adopt longitudinal designs and more diverse samples to validate and generalize the observed patterns, as well as explore potential moderators and heterogeneity in teachers’ adoption behaviors (Laursen and Hoff, 2006).

The study makes a theoretical contribution by extending EVT to a novel technology adoption context in higher education and by demonstrating the differential roles of motivational beliefs in predicting intention versus enacted behavior. Specifically, the dominance of Utility Value over Intrinsic Value observed in our sample invites a cultural interpretation through the lens of “Pragmatic Reason” (shiyong lixing) (Rošker, 2020). Unlike Western educational contexts that often prioritize personal interest or autonomous enjoyment in achievement (Tao and Hong, 2014), the Chinese philosophical tradition emphasizes the instrumental application of wisdom to solve actual problems for survival and continuity (Rošker, 2020). This cultural schema predisposes faculty to view Gen AI primarily as a pragmatic tool for coping with performance pressures (Utility), rather than an object of curiosity (Intrinsic).

Practically, these findings necessitate a strategic shift in professional development. Rather than generic workshops focusing on the technical novelty of Gen AI, institutions should design “pain-point oriented” training. Initiatives must explicitly demonstrate how Gen AI reduces specific administrative burdens and accelerates research output to meet performance metrics, thereby appealing directly to the faculty’s need for utility. Furthermore, given the critical role of self-efficacy, training should move beyond instruction to offer “mastery experiences,” where faculty facilitate small but immediate wins in their daily workflow to overcome technical anxiety.

Future research should expand on these insights by adopting cross-cultural comparative designs. Researchers should test whether the “Utility-first” pattern is unique to cultures characterized by pragmatic rationality or social-oriented achievement (Tao and Hong, 2014), versus cultures where intrinsic interest is a stronger predictor. Additionally, drawing on Zhu et al. (2023), future scholarship should explore the “paradoxical expectations” of Gen AI—balancing its utility as a tool against the anxiety of replacement—using longitudinal designs to track whether the pragmatic adoption observed here evolves into intrinsic interest as faculty achieve proficiency.

Nonetheless, the study has limitations as well; these include its cross-sectional design, which precludes causal inference, and the restricted representativeness of the sample, drawn from only two universities in one province of China. Future studies should employ more diverse samples to validate and generalize the observed patterns, as well as explore potential moderators and heterogeneity in teachers’ adoption behaviors (Laursen and Hoff, 2006). The actual frequency of Gen AI usage was assessed via a single-item measure. While single-item scales are frequently employed in behavioral research to reduce respondent burden and exhibit high face validity for concrete, objective behaviors, they may lack the psychometric robustness and internal consistency associated with multi-item inventories. This methodological constraint could potentially influence the reliability estimates within the SEM framework. Future research should consider adopting multi-dimensional behavioral scales to further cross-validate the structural paths identified in this study. Finally, given that the AVE values for certain adapted subscales (e.g., Gen AI Cost) were marginally acceptable, future scholarship should prioritize the development and validation of more culturally sensitive measurement instruments that capture the nuances of Gen AI adoption in the Chinese educational context with greater precision.

## Data Availability

The raw data supporting the conclusions of this article will be made available by the authors, without undue reservation.
