# Supplementary material for: Expectancy and value beliefs predicting generative AI use: evidence from Chinese university faculty
Source: Front Psychol. 2026 Apr 24;17:1758074. doi: 10.3389/fpsyg.2026.1758074 (PMC13153113; doi:10.3389/fpsyg.2026.1758074)
Supplement: Supplementary file 1 [file Supplementary_file_1.docx]

**Appendix A Survey of Instructor Engagement with Generative AI (Gen AI)**

**1. What is your age?** [Fill in the blank]

**2. Your gender:** [Single Choice] * | ○ Male | ○ Female |

**3. What is your position type at the university?** [Single Choice] | ○ Teaching and Research Faculty | | ○ Full-time Research Faculty (Researchers primarily engaged in scientific research) | | ○ Full-time Teaching Faculty (Teachers primarily engaged in teaching) | | ○ Counselor (Teachers responsible for students' ideological and political education and management) | | ○ Administrative Staff (Personnel engaged in administrative management, discipline construction, or teaching management) | | ○ Laboratory/Technical Staff (Personnel engaged in experimental teaching, laboratory management, or technical support) | | ○ Other (Please specify) _________________ |

**4. What is your professional title?** [Single Choice] * | ○ Junior | | ○ Intermediate | | ○ Senior |

**5. As of today, how many years have you worked as a university teacher?** [Single Choice] * | ○ Less than 5 years | | ○ 6-10 years | | ○ 11-20 years | | ○ 21-30 years | | ○ More than 30 years |

**【Gen AI Task Values & Gen AI Cost. Intrinsic Value: 1-5; Utility Value: 6-10; Attainment Value: 11-16; 2.2.3 Gen AI Cost: 17-21】6. Below are several statements. To what extent does each statement describe your feelings, thoughts, or practices regarding the use of Gen AI (e.g., Deepseek, Kimi, etc.) in your work? Please answer truthfully. There are no right or wrong answers. For each statement, please select one of the seven options.** [Matrix Single Choice] *

*Scale Anchors:* 1 = Strongly Disagree 2 = Disagree 3 = Somewhat Disagree 4 = Neutral 5 = Somewhat Agree 6 = Agree 7 = Strongly Agree

| **Items** | **1** | **2** | **3** | **4** | **5** | **6** | **7** |
| --- | --- | --- | --- | --- | --- | --- | --- |
| 1) I find using Gen AI for teaching very interesting. | ○ | ○ | ○ | ○ | ○ | ○ | ○ |
| 2) I feel that using Gen AI allows me to derive pleasure from teaching. | ○ | ○ | ○ | ○ | ○ | ○ | ○ |
| 3) I feel that using Gen AI in my work brings me a sense of accomplishment and fun. | ○ | ○ | ○ | ○ | ○ | ○ | ○ |
| 4) I enjoy the fun of using Gen AI for scientific research. | ○ | ○ | ○ | ○ | ○ | ○ | ○ |
| 5) I like exploring how to apply Gen AI to my teaching. | ○ | ○ | ○ | ○ | ○ | ○ | ○ |
| 6) Using Gen AI helps me complete teaching tasks more efficiently. | ○ | ○ | ○ | ○ | ○ | ○ | ○ |
| 7) I feel Gen AI is very useful for completing my research tasks. | ○ | ○ | ○ | ○ | ○ | ○ | ○ |
| 8) I feel Gen AI helps alleviate my teaching burden. | ○ | ○ | ○ | ○ | ○ | ○ | ○ |
| 9) I feel Gen AI helps improve my work efficiency. | ○ | ○ | ○ | ○ | ○ | ○ | ○ |
| 10) I believe Gen AI can improve my efficiency in grading student assignments. | ○ | ○ | ○ | ○ | ○ | ○ | ○ |
| 11) Using Gen AI will make my career more successful. | ○ | ○ | ○ | ○ | ○ | ○ | ○ |
| 12) It is important for me to use Gen AI in my work. | ○ | ○ | ○ | ○ | ○ | ○ | ○ |
| 13) Being able to use Gen AI in teaching makes me feel proud. | ○ | ○ | ○ | ○ | ○ | ○ | ○ |
| 14) If I can use Gen AI to assist in my research, I would feel more competent as a university teacher. | ○ | ○ | ○ | ○ | ○ | ○ | ○ |
| 15) In my work, using Gen AI gives me a greater sense of achievement. | ○ | ○ | ○ | ○ | ○ | ○ | ○ |
| 16) Using Gen AI for teaching makes me feel that my teaching ability is higher. | ○ | ○ | ○ | ○ | ○ | ○ | ○ |
| 【Gen AI Cost】17) Using Gen AI in teaching or research requires investing too much time. | ○ | ○ | ○ | ○ | ○ | ○ | ○ |
| 【Gen AI Cost】18) Learning Gen AI costs me too much time and energy. | ○ | ○ | ○ | ○ | ○ | ○ | ○ |
| 【Gen AI Cost】19) I have too many other matters to handle and cannot invest enough energy to learn and use Gen AI. | ○ | ○ | ○ | ○ | ○ | ○ | ○ |
| 【Gen AI Cost】20) Using Gen AI makes me feel very frustrated. | ○ | ○ | ○ | ○ | ○ | ○ | ○ |
| 【Gen AI Cost】21) Using Gen AI in my work requires investing excessive effort. | ○ | ○ | ○ | ○ | ○ | ○ | ○ |

**【Gen AI Self-Efficacy】7. Please indicate your level of confidence in applying Gen AI technology in your work. Please use the scale of 1-7 to indicate how much you agree with the following descriptions of your confidence in using AIGC (AI-Generated Content) technology.** [Matrix Single Choice] *

*Scale Anchors:* 1 = Strongly Disagree 2 = Disagree 3 = Somewhat Disagree 4 = Neutral 5 = Somewhat Agree 6 = Agree 7 = Strongly Agree

| **Items** | **1** | **2** | **3** | **4** | **5** | **6** | **7** |
| --- | --- | --- | --- | --- | --- | --- | --- |
| 1) I am fully confident in my ability to use Gen AI technology to assist with research work. | ○ | ○ | ○ | ○ | ○ | ○ | ○ |
| 2) I am fully confident in my ability to use Gen AI technology to enhance teaching quality. | ○ | ○ | ○ | ○ | ○ | ○ | ○ |
| 3) I am fully confident in my ability to use Gen AI technology to create attractive PPTs or course resources. | ○ | ○ | ○ | ○ | ○ | ○ | ○ |
| 4) I am fully confident in my ability to use Gen AI technology to assist with administrative work (e.g., drafting materials, organizing data). | ○ | ○ | ○ | ○ | ○ | ○ | ○ |
| 5) I am fully confident in my ability to use AIGC technology to search for, integrate, and analyze data. | ○ | ○ | ○ | ○ | ○ | ○ | ○ |

**【Gen AI Behavioral Intention】8. To what extent do you agree with the following statements regarding your thoughts on using Gen AI?** [Matrix Single Choice] *

*Scale Anchors:* 1 = Strongly Disagree 2 = Disagree 3 = Somewhat Disagree 4 = Neutral 5 = Somewhat Agree 6 = Agree 7 = Strongly Agree

| **Items** | **1** | **2** | **3** | **4** | **5** | **6** | **7** |
| --- | --- | --- | --- | --- | --- | --- | --- |
| 1) I intend to continue using Gen AI tools in the future. | ○ | ○ | ○ | ○ | ○ | ○ | ○ |
| 2) I will try to use Gen AI tools in my daily life/work. | ○ | ○ | ○ | ○ | ○ | ○ | ○ |
| 3) I plan to continue using Gen AI tools. | ○ | ○ | ○ | ○ | ○ | ○ | ○ |

**【Frequency of Gen AI usage】9. Do you currently use Gen AI technology in your work (administration, teaching, research, etc.)?** [Matrix Single Choice] *

*Scale:* 1 = Never use ... 10 = Use multiple times every day

|  | **1 Never use** | **2** | **3** | **4** | **5** | **6** | **7** | **8** | **9** | **10 Use multiple times every day** |
| --- | --- | --- | --- | --- | --- | --- | --- | --- | --- | --- |
| Usage Status | ○ | ○ | ○ | ○ | ○ | ○ | ○ | ○ | ○ | ○ |
