# Supplementary material for: Expectancy and value beliefs predicting generative AI use: evidence from Chinese university faculty
Source: Front Psychol. 2026 Apr 24;17:1758074. doi: 10.3389/fpsyg.2026.1758074 (PMC13153113; doi:10.3389/fpsyg.2026.1758074)
Supplement: Supplementary file 2 [file Supplementary_file_2.DOCX]

**Appendix B Item-level Correlations**

|  | 1 | 2 | 3 | 4 | 5 | 6 | 7 | 8 | 9 | 10 | 11 | 12 | 13 | 14 | 15 | 16 | 17 | 18 | 19 | 20 | 21 | 22 | 23 | 24 | 25 | 26 | 27 | 28 | 29 | 30 |
| --- | --- | --- | --- | --- | --- | --- | --- | --- | --- | --- | --- | --- | --- | --- | --- | --- | --- | --- | --- | --- | --- | --- | --- | --- | --- | --- | --- | --- | --- | --- |
| 1 Intrinsic_Item1 | 1 |  |  |  |  |  |  |  |  |  |  |  |  |  |  |  |  |  |  |  |  |  |  |  |  |  |  |  |  |  |
| 2 Intrinsic_Item2 | **.55** | 1 |  |  |  |  |  |  |  |  |  |  |  |  |  |  |  |  |  |  |  |  |  |  |  |  |  |  |  |  |
| 3 Intrinsic_Item3 | **.45** | **.48** | 1 |  |  |  |  |  |  |  |  |  |  |  |  |  |  |  |  |  |  |  |  |  |  |  |  |  |  |  |
| 4 Intrinsic_Item4 | **.49** | **.53** | **.49** | 1 |  |  |  |  |  |  |  |  |  |  |  |  |  |  |  |  |  |  |  |  |  |  |  |  |  |  |
| 5 Intrinsic_Item5 | **.52** | **.46** | **.49** | **.50** | 1 |  |  |  |  |  |  |  |  |  |  |  |  |  |  |  |  |  |  |  |  |  |  |  |  |  |
| 6 Utility_Item1 | **.36** | **.29** | **.29** | **.27** | **.32** | 1 |  |  |  |  |  |  |  |  |  |  |  |  |  |  |  |  |  |  |  |  |  |  |  |  |
| 7 Utility_Item2 | **.30** | **.33** | **.29** | **.29** | **.37** | **.54** | 1 |  |  |  |  |  |  |  |  |  |  |  |  |  |  |  |  |  |  |  |  |  |  |  |
| 8 Utility_Item3 | **.29** | **.30** | **.28** | **.30** | **.31** | **.56** | **.54** | 1 |  |  |  |  |  |  |  |  |  |  |  |  |  |  |  |  |  |  |  |  |  |  |
| 9 Utility_Item4 | **.35** | **.26** | **.33** | **.27** | **.35** | **.56** | **.59** | **.58** | 1 |  |  |  |  |  |  |  |  |  |  |  |  |  |  |  |  |  |  |  |  |  |
| 10 Utility_Item5 | **.27** | **.22** | **.26** | **.32** | **.22** | **.48** | **.56** | **.58** | **.54** | 1 |  |  |  |  |  |  |  |  |  |  |  |  |  |  |  |  |  |  |  |  |
| 11 Utility_Item6 | **.30** | **.24** | **.32** | **.32** | **.36** | **.54** | **.89** | **.55** | **.60** | **.58** | 1 |  |  |  |  |  |  |  |  |  |  |  |  |  |  |  |  |  |  |  |
| 12 Attainment_Item1 | **.32** | **.28** | **.23** | **.23** | **.29** | **.36** | **.41** | **.34** | **.37** | **.38** | **.39** | 1 |  |  |  |  |  |  |  |  |  |  |  |  |  |  |  |  |  |  |
| 13 Attainment_Item2 | **.38** | **.36** | **.31** | **.29** | **.33** | **.32** | **.32** | **.39** | **.43** | **.28** | **.35** | **.49** | 1 |  |  |  |  |  |  |  |  |  |  |  |  |  |  |  |  |  |
| 14 Attainment_Item3 | **.39** | **.34** | **.31** | **.34** | **.41** | **.31** | **.33** | **.30** | **.36** | **.31** | **.38** | **.47** | **.60** | 1 |  |  |  |  |  |  |  |  |  |  |  |  |  |  |  |  |
| 15 Attainment_Item4 | **.36** | **.37** | **.26** | **.31** | **.36** | **.31** | **.35** | **.32** | **.34** | **.33** | **.33** | **.57** | **.60** | **.56** | 1 |  |  |  |  |  |  |  |  |  |  |  |  |  |  |  |
| 16 Attainment_Item5 | **.31** | **.32** | **.30** | **.25** | **.33** | **.34** | **.33** | **.39** | **.41** | **.36** | **.40** | **.52** | **.59** | **.57** | **.52** | 1 |  |  |  |  |  |  |  |  |  |  |  |  |  |  |
| 17 Cost_Item1 | **-.22** | **-.24** | **-.22** | **-.16** | **-.22** | **-.20** | **-.23** | **-.20** | **-.22** | **-.24** | **-.31** | **-.23** | **-.3** | **-.29** | **-.32** | **-.25** | 1 |  |  |  |  |  |  |  |  |  |  |  |  |  |
| 18 Cost_Item2 | **-.28** | **-.22** | **-.23** | **-.16** | **-.27** | **-.22** | **-.25** | **-.26** | **-.24** | **-.21** | **-.24** | **-.16** | **-.32** | **-.29** | **-.20** | **-.27** | **.45** | 1 |  |  |  |  |  |  |  |  |  |  |  |  |
| 19 Cost_Item3 | **-.30** | **-.27** | **-.25** | **-.21** | **-.32** | **-.27** | **-.27** | **-.25** | **-.29** | **-.28** | **-.33** | **-.24** | **-.30** | **-.31** | **-.32** | **-.23** | **.48** | **.46** | 1 |  |  |  |  |  |  |  |  |  |  |  |
| 20Cost_Item4 | **-.28** | **-.23** | **-.23** | **-.22** | **-.25** | **-.34** | **-.27** | **-.32** | **-.35** | **-.23** | **-.32** | **-.27** | **-.32** | **-.31** | **-.27** | **-.28** | **.42** | **.51** | **.46** | 1 |  |  |  |  |  |  |  |  |  |  |
| 21Cost_Item5 | **-.24** | **-.19** | **-.23** | **-.19** | **-.22** | **-.19** | **-.27** | **-.20** | **-.27** | **-.21** | **-.27** | **-.24** | **-.29** | **-.29** | **-.30** | **-.28** | **.48** | **.48** | **.49** | **.44** | 1 |  |  |  |  |  |  |  |  |  |
| 22Efficacy_Item1 | **.36** | **.37** | **.32** | **.26** | **.33** | **.34** | **.37** | **.37** | **.32** | **.33** | **.38** | **.35** | **.40** | **.38** | **.36** | **.40** | **-.27** | **-.29** | **-.31** | **-.29** | **-.30** | 1 |  |  |  |  |  |  |  |  |
| 23Efficacy_Item2 | **.41** | **.36** | **.30** | **.29** | **.33** | **.39** | **.35** | **.36** | **.33** | **.27** | **.28** | **.34** | **.42** | **.37** | **.34** | **.37** | **-.18** | **-.22** | **-.19** | **-.33** | **-.24** | **.53** | 1 |  |  |  |  |  |  |  |
| 24Efficacy_Item3 | **.30** | **.31** | **.33** | **.37** | **.37** | **.26** | **.29** | **.24** | **.31** | **.27** | **.33** | **.34** | **.36** | **.42** | **.36** | **.38** | **-.27** | **-.19** | **-.24** | **-.27** | **-.26** | **.46** | **.38** | 1 |  |  |  |  |  |  |
| 25Efficacy_Item4 | **.40** | **.31** | **.33** | **.31** | **.31** | **.33** | **.31** | **.29** | **.36** | **.38** | **.36** | **.33** | **.39** | **.38** | **.34** | **.35** | **-.29** | **-.22** | **-.28** | **-.29** | **-.29** | **.53** | **.52** | **.46** | 1 |  |  |  |  |  |
| 26Efficacy_Item5 | **.38** | **.35** | **.30** | **.33** | **.36** | **.33** | **.37** | **.34** | **.32** | **.29** | **.32** | **.34** | **.38** | **.34** | **.36** | **.42** | **-.22** | **-.20** | **-.26** | **-.27** | **-.29** | **.51** | **.49** | **.41** | **.49** | 1 |  |  |  |  |
| 27Intention_Item1 | **.38** | **.32** | **.32** | **.31** | **.35** | **.37** | **.40** | **.36** | **.32** | **.31** | **.32** | **.39** | **.35** | **.40** | **.40** | **.42** | **-.28** | **-.24** | **-.25** | **-.26** | **-.27** | **.34** | **.40** | **.32** | **.35** | **.35** | 1 |  |  |  |
| 28Intention_Item2 | **.41** | **.33** | **.30** | **.34** | **.28** | **.37** | **.42** | **.36** | **.38** | **.39** | **.36** | **.36** | **.33** | **.35** | **.33** | **.34** | **-.27** | **-.23** | **-.28** | **-.28** | **-.28** | **.39** | **.36** | **.27** | **.37** | **.35** | **.59** | 1 |  |  |
| 29Intention_Item3 | **.32** | **.28** | **.24** | **.26** | **.27** | **.37** | **.35** | **.33** | **.32** | **.29** | **.28** | **.32** | **.30** | **.30** | **.35** | **.32** | **-.22** | **-.20** | **-.27** | **-.30** | **-.23** | **.36** | **.37** | **.24** | **.33** | **.32** | **.60** | **.65** | 1 |  |
| 30Gen AI Usage Frequency | .12 | .10 | .02 | .05 | -.01 | .08 | .09 | .07 | .07 | .08 | .04 | .07 | **.11** | **.12** | .03 | **.14** | -.08 | -.10 | .02 | -.06 | **-.16** | **.14** | **.18** | .10 | **.15** | **.14** | .08 | .02 | .01 | 1 |
